# Artemisinin-induced parasite dormancy: a plausible mechanism for treatment failure

**DOI:** 10.1186/1475-2875-10-56

**Published:** 2011-03-08

**Authors:** Andrea Codd, Franka Teuscher, Dennis E Kyle, Qin Cheng, Michelle L Gatton

**Affiliations:** 1 Malaria Drug Resistance and Chemotherapy Laboratory, Queensland Institute of Medical Research, Brisbane, Australia; 2 Drug Resistance and Diagnostics, Australian Army Malaria Institute, Brisbane, Australia; 3 Department of Global Health, College of Public Health, University of South Florida, USA

## Abstract

**Background:**

Artemisinin-combination therapy is a highly effective treatment for uncomplicated falciparum malaria but parasite recrudescence has been commonly reported following artemisinin (ART) monotherapy. The dormancy recovery hypothesis has been proposed to explain this phenomenon, which is different from the slower parasite clearance times reported as the first evidence of the development of ART resistance.

**Methods:**

In this study, an existing *P. falciparum *infection model is modified to incorporate the hypothesis of dormancy. Published *in vitro *data describing the characteristics of dormant parasites is used to explore whether dormancy alone could be responsible for the high recrudescence rates observed in field studies using monotherapy. Several treatment regimens and dormancy rates were simulated to investigate the rate of clinical and parasitological failure following treatment.

**Results:**

The model output indicates that following a single treatment with ART parasitological and clinical failures occur in up to 77% and 67% of simulations, respectively. These rates rapidly decline with repeated treatment and are sensitive to the assumed dormancy rate. The simulated parasitological and clinical treatment failure rates after 3 and 7 days of treatment are comparable to those reported from several field trials.

**Conclusions:**

Although further studies are required to confirm dormancy *in vivo*, this theoretical study adds support for the hypothesis, highlighting the potential role of this parasite sub-population in treatment failure following monotherapy and reinforcing the importance of using ART in combination with other anti-malarials.

## Background

Artemisinin-combination therapy (ACT) is the WHO recommended treatment for uncomplicated *Plasmodium falciparum *malaria [1]. It is imperative that artemisinin (ART) is used in combination with other anti-malarials partially because parasite recrudescence is common following ART monotherapy. The reported 28-day parasitological failure rates of ART monotherapy vary widely, from 2% to 50% [2,3]. This appears to be a feature of ART monotherapy and is independent of the observation of increased parasite clearance times which is considered the first evidence of ART resistance in the parasite [4]. It had been thought that because the ART class of compounds have a very short half-life they cannot eliminate all parasites during the short treatment time. However, while the rate of parasite recrudescence generally decreases with increasing duration of treatment, parasite recrudescence following 5 or 7 days of treatment is still common [3,5], and is hard to explain based on the drugs' potency and pharmacokinetic/pharmocodynamic properties alone. Furthermore, the treatment failures observed after ART monotherapy are not due to parasite resistance since retreatment with the same compound is equally effective as the initial treatment [6,7].

Another hypothesis that has been presented for the frequent occurrence of recurrent infections following ART monotherapy is that some ART-treated parasites enter a state of quiescence, or dormancy, where they are protected from the drugs lethal effects, but recover at a later date to resume normal growth [8,9]. Recent *in vitro *studies show that dormancy can be readily induced in *P. falciparum *using dihydroartemisinin (DHA) [10] or ART itself [11]. Following exposure to 200 ng/ml (~7 × 10^-7 ^M) DHA for 6 hours parasites recovered between 3 and 20 days post-treatment at an overall rate between 0.044% and 0.145%, depending on the parasite line [10]. Complete eradication of parasites was only achieved when a threshold concentration of 3 × 10^-8 ^M of ART was administered twice daily for at least five days or when used in combination with mefloquine at levels above the respective minimum inhibitory concentrations (MICs) [12]. Interestingly this dormancy state has also been postulated to occur following *in vitro *treatment with other anti-malarial drugs such as pyrimethamine, atovaquone and mefloquine [13-15], although high recrudescence rates following monotherapy with these drugs (that were not due to the selection of a parasite subpopulation carrying resistance mutations) have not been commonly reported. This may be due to the long half-lives of these drugs compared to ART.

With the growing reliance on ACT, but only some combinations available as fixed-dose treatments, the risk of ART monotherapy is ever present so it is important to investigate whether dormancy is induced *in vivo *and, if so, the impact that it may have on the successful treatment of patients. This study is focused on the theoretical question of whether dormancy could be responsible for the high recrudescence rates observed in field studies using monotherapy, under the assumption that the *in vitro *findings are applicable *in vivo*. The process being modelled is not one of parasite resistance, but a drug-induced temporary pause in the development of some parasites.

Hoshen *et al *[9] have previously modelled the concept of dormancy using a PK-PD model for 7 days following treatment. However, the parameters used to characterize dormancy, 0.2% of parasites becoming dormant for 18 hours, significantly under-estimate the duration of dormancy compared to recent data [10]. Here the most recent and comprehensive data on dormancy rates and duration are used in an in-host stochastic simulation model to better understand the potential impact of parasite dormancy.

## Methods

A previously published computer simulation model of the with-in host dynamics of *P. falciparum *infections was expanded to incorporate parasite dormancy [16] (see Additional file 1). The model included three types of human immune response which develop during an infection, as well as antigenic variation within the parasite. The parameter values used were those obtained previously from fitting the model to data from malaria naïve individuals infected with the El Limon and Santee Cooper *P. falciparum *strains.

The model was used to simulate treatment using a daily dose of ART given for 1, 3 or 7 days. The treatment is initiated the day after the first occurrence of fever (Day 1), or up to 3 days later (Days 2 to 4). This delay in treatment was included to reflect the reported treatment seeking behaviour of individuals where only a small proportion of people received treatment within 24 hours of the onset of symptoms [17-19]. Since the occurrence of fever is assumed to be associated with the release of toxins from rupturing infected erythrocytes (iRBC), the predominant parasite stage during the fever is ring-stage. Therefore parasites are mature-stages when treatment is administered on Days 1 and 3, and ring-stages when treatment is administered on Days 2 and 4.

In order to include dormancy in the model it was assumed that ART treatment caused a small percentage of asexual parasites to become dormant (to later recover) and killed the remainder. Inadequate killing of parasites by the drug was not considered. The dormant parasites wake at the same asexual stage, expressing the same PfEMP1 variant, as when treatment was administered. It was assumed the drug had a stage-specific effect with a higher proportion of ring-stage parasites becoming dormant than mature stage parasites [8,11]. The dormancy rates and distribution of waking times for ring-stage parasites reflected *in vitro *data following parasite exposure to 200 ng/ml DHA [10]. For the single dose treatment, the majority of the parasites were assumed to be killed by the treatment and dormancy rates were 0.1158% for rings and 0.050% for mature stages, with recovery to resume active growth occurring between 4 and 17 days post-treatment [10]. For the three-day regime the dormancy rate of ring-stage parasites was assumed to be 0.0116%, 10-fold lower than the single dose treatment [10]. Due to a lack of experimental data relating to recovery after seven days of dosing two sets of simulations were conducted; 1) using the same overall dormancy rate as for the three-day simulation, and 2) a regime where overall recovery was 0.0012%, or approximately 100-fold lower than for a single dose. In all multiple dosing simulations the waking profile and proportion of mature stages becoming dormant were unchanged from the single dose simulation, and treatment was applied when parasites were ring-stage.

For each treatment regime considered 2,500 simulations of infections in malaria naïve hosts were conducted. The primary output in this study was the proportion of simulations in which the individual failed treatment, that is, had clinical symptoms re-develop following treatment, and the timing of these symptoms. The 28-day parasitological failure rates were also reported.

## Results

### Host immunity and treatment failure

**Figure 1:**
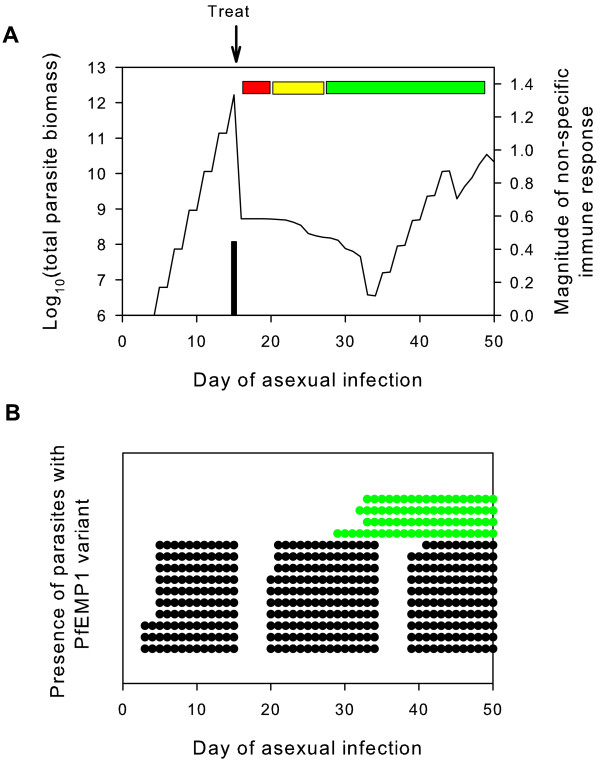
**Example of simulated parasite dynamics following treatment with a single dose of ART and 0 day treatment delay**. A) Within-host parasite density over the first 50 days of infection and corresponding non-specific immune response (black bar). The coloured horizontal bars indicate three distinct phases post-treatment: dormancy (red), recovery (yellow) and renewed growth (green). B) Presence/absence of PfEMP1 variants that have > 10,000 parasites expressing the variant. Black: PfEMP1 variants where the corresponding anti-PfEMP1 antibodies were triggered prior to treatment. Green: PfEMP1 variants where corresponding anti-PfEMP1 antibodies were not triggered prior to treatment (ie density for parasites expressing variant prior to treatment did not reach the antibody threshold of 12 parasites/μl).

The results show that there were three distinct phases in parasite dynamics following treatment (Figure 1). The first represented the minimum dormancy period immediately following treatment when all surviving parasites are dormant. The second phase occurred after the parasites started to recover from dormancy. During this period the anti-PfEMP1 antibodies triggered prior to treatment were able to control parasitaemia, causing a gradual decrease in the absolute number of viable (dormant or growing) parasites. The final phase was one of renewed growth which occurred when parasites switched to a PfEMP1 variant for which no antibodies had been produced.

### Recrudescence rate and timing following a single dose of ART

**Figure 2:**
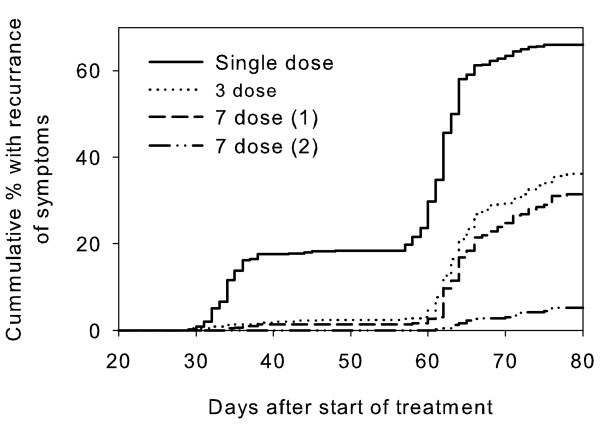
**Profile of simulated clinical failures following treatment with artemisinin for single and multiple dosing regimes**. Overall dormancy rates for ring-stage parasites were 0.1158% for single dose, 0.0116% for 3 dose and 7 dose (1), and 0.0012% for 7 dose (2).

The overall simulated clinical failure rates were 60.6%, 67.0%, 45.2% and 62.6% for treatment given 1 to 4 days after the initial fever episode, respectively. Symptomatic recurrence occurred at two distinct time periods: 29 to 48 days after the start of treatment and 55 to 78 days after the start of treatment (Figure 2). The clinical failure rate during the first period varied between 4.8% (for treatment given on Day 3) and 17.2% (for treatment given on Day 2). The majority of failures occurred in the later group between 55 and 78 days.

The proportion of the simulations that had a biomass equivalent to > 50 parasites/μl at 28 days (parasitological failures) after treatment were 58.2%, 77.2%, 28.2% and 39.5% for treatment given on Days 1 to 4, respectively. Although patent parasitaemia was commonly seen in the simulations at 28-days post-treatment, the majority of these infections were suppressed below the pyrogenic threshold by the immune system during the first 48 days, creating a difference in the parasitological and clinical failure rates.

The impact of the waking profile on treatment failure rates was investigated. Earlier recovery of parasites from dormancy resulted in higher overall failure rates, earlier presentation of failures and a larger proportion of failures occurring before 50 days.

### Multiple ART dosing

**Figure 3:**
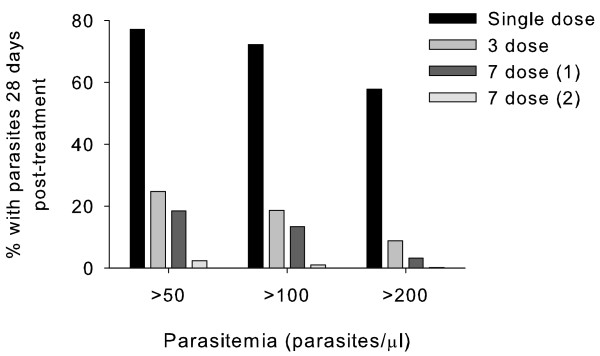
**Predicted parasitological failure rates at 28 days post-treatment for several artemisinin dosing regimes**. Failure rates for three limits of detection are displayed. Dormancy rates are as described for Figure 2.

The proportion of treatment failures was highly sensitive to the dosing regimen and dormancy rate. However the relationship between clinical failure rate and overall dormancy rate was not linear. The proportion of clinical failures decreased from 67.0% after a single ART dose (overall dormancy rate of 0.1158%) to 37.8% after 3 doses (overall dormancy rate of 0.0116%; Figure 2). In comparison the proportion of simulations with parasitological failures (> 50 parasites/μl) at 28 days post-treatment showed a larger proportional decrease from 77.2% to 24.7% (Figure 3). When the dosing was extended to seven days there was a slight, but not significant, reduction in clinical and parasitological failure compared to the three-day dose when the three-dose dormancy rates were applied (Figure 2). The clinical failure and 28-day parasitological failure rates dropped dramatically to 5.2% and 1.0% respectively when the dormancy rate was decreased to 0.0012% for the 7-day treatment (Figure 3).

## Discussion

*In vitro *data indicate that exposure to physiologically-relevant concentrations of the ART class of drugs can induce a period of dormancy in *P. falciparum *parasites [10,11]. While this finding is yet to be confirmed *in vivo*, the current modelling study was designed to investigate the impact that dormancy alone could have on treatment outcome. The results suggest that the dormancy recovery hypothesis can explain the unusually high treatment failure rates observed following ART monotherapy. It should be noted that this study does not address the issue of inadequate killing by the drug, which is an alternate hypothesis for the observed treatment failures.

Several treatment regimens were simulated: a single dose treatment, a three-day treatment and a seven-day treatment. Not surprisingly, the treatment failure rates decreased with longer treatment due to the lower assumed dormancy rate. The clinical failures were predicted to occur in two distinct groups with the proportion in each group dependent on the dormancy recovery rate; lower dormancy recovery rates were associated with a higher proportion of late failures. This pattern most probably reflects the interaction of the waking parasites with the host immune system.

All of the simulated infections had some recrudescent parasites due to waking dormant parasites. However, only a percentage of these developed into clinical recurrences or a measurable parasitaemia due to the involvement of the immune system. The presence of recrudescent parasites (detected by PCR) without clinical signs or symptoms was noted in a study conducted in children in Gabon and was attributed to humoral immunity being able to control low-level parasitemia [20]. It is important to note that although the model used in the current study represented malaria naïve hosts, it does allow the development of some immunity to the specific parasite through the duration of the infection. It would be expected that as the host immune response improves with repeated exposure, fewer recrudescent infections would develop.

The predicted 28-day parasitological failure rate in the simulated population following three days of treatment agrees with the rates reported from several clinical trials. In the simulated output 25% of infections had > 50 parasites/μl at day 28 compared to 29% in a Thai study of hospitalised adults where failure was determined by intensive microscopy [7] and 28% (PCR-corrected) in a study in symptomatic children in Gabon [20]. This suggests that the dormancy rates taken from *in vitro *data following three short exposures to DHA may be representative of the *in vivo *dormancy rate. In lieu of data on the dormancy rate following repeated dosing akin to seven days of treatment, but recognizing that dormancy parameters are dose related [10], two different simulations for seven-day treatment were conducted using dormancy rates of 0.0116% and 0.0012%. The predicted 28-day parasitological failure rates for these simulations were 18.4% and 2.4%, respectively. In the Central African Republic the 28-day PCR-corrected failure rate in non-immune adults following a seven-day course of artesunate treatment was 5% [21], suggesting the *in vivo *dormancy recovery rate following seven-day treatment may be close to 0.0012%.

Differences were noted in the predicted treatment failure rate when the profile of recovery changed. Although parasite recovery was permitted for up to 17 days, in terms of recrudescence, it is the first brood of parasites to wake after treatment ceases which have the largest impact. The waking profiles used in this study did not allow parasites to start to recover until three days after treatment. It is possible that parasites recover earlier than this but this is expected to have minimal impact on the model results since these parasites would be re-exposed to the drug during this period due to multiple dosing regimes.

The timing of treatment also impacted on the predicted treatment failure rates. There were differences between treatments on odd or even days, a result related to whether the parasites were treated at ring-stage or mature stages. Although not well tested, there are preliminary laboratory data [11](Kyle, unpublished data) and modelling [22] which indicates that mature-stage parasites are more sensitive to ART, leading to the assumption of a lower dormancy rate for mature parasites. Hence, a single dose applied to mature stage parasites in the model produced lower failure rates than a single dose administered to ring-stage parasites. However, further laboratory studies are required to better understand the effect of ART on mature stage parasites, particularly the effect of multiple dosing.

This study has demonstrated that inclusion of ART-induced dormancy in a within-host simulation model of *P. falciparum *can generate treatment failures, and that the predicted proportion of treated hosts with detectable parasites 28 days after treatment is generally comparable to those measured in ART monotherapy field trials. Further clinical investigations are required to confirm the occurrence of dormancy *in vivo*, but this theoretical study adds support for the dormancy recovery hypothesis, and highlights the potential role this parasite sub-population may have in treatment failure. When deployed in ACT, the impact of the dormant population on efficacy is likely reduced by the companion drug lowering the dormancy rate [10] and, in the case of long-acting companions, mopping up the waking parasites. A better understanding of the dormancy recovery process and the impact that companion drugs have on dormant and recovered parasites will help establish treatment combinations and regimes that minimize the possibility of dormant parasites surviving to cause treatment failure. This improved understanding of ART-induced dormancy needs to occur in parallel to the efforts being made to understand and contain the development of ART resistance since these focus on different, but important, aspects of the parasites response to ART; dormancy is proposed to be an inherent parasite response following exposure to ART, while resistance involves adaptation or changes of the parasite's response to ART, particularly the susceptibility of ring-stage parasites [22].

## Conclusions

Artemisinin induced dormancy provides one plausible explanation for the high level of recrudescence reported in the field following monotherapy. Although repeated treatment reduced the dormancy recovery rate and the rate of recrudescence, a small proportion of failures were still predicted. The findings highlight the importance of using artemisinin in combination with other anti-malarial drugs and using the combinations as fixed-dose treatment.

## Competing interests

The authors declare that they have no competing interests.

## Authors' contributions

AC conducted the programming and computational work and drafted the manuscript. FT helped draft the manuscript. DEK contributed to conceiving the study. QC contributed to conceiving the study and helped draft the manuscript. MLG participated in conceiving and designing the study, analyzed some results and helped draft the manuscript. All authors read and approved the final manuscript.

## Supplementary Material

Additional file 1 **Details of within-host model of *P. falciparum *infection in naïve host**. Click here for file

## References

[B1] World Health Organization Guidelines for the treatment of malaria 2010 2 Geneva 25473692

[B2] Meshnick SR Taylor TE Kamchonwongpaisan S Artemisinin and the antimalarial endoperoxides: from herbal remedy to targeted chemotherapy Microbiol Rev 1996 60 301 315 8801435 10.1128/mr.60.2.301-315.1996 PMC239445

[B3] McIntosh HM Olliaro P Artemisinin derivatives for treating uncomplicated malaria Cochrane Database Syst Rev 1999 2 CD000256 10.1002/14651858.CD000256 PMC6532741 10796519

[B4] Dondorp AM Nosten F Yi P Das D Phyo AP Tarning J Lwin KM Ariey F Hanpithakpong W Lee SJ Ringwald P Silamut K Imwong M Chotivanich K Lim P Herdman T An SS Yeung S Singhasivanon P Day NPJ Lindegardh N Socheat D White NJ Artemisinin resistance in Plasmodium falciparum malaria N Engl J Med 2009 361 5 455 467 10.1056/NEJMoa0808859 19641202 PMC3495232

[B5] Giao PT Binh TQ Kager PA Long HP Van Thang N Van Nam N de Vries PJ Artemisinin for treatment of uncomplicated falciparum malaria: is there a place for monotherapy? Am J Trop Med Hyg 2001 65 690 695 11791958 10.4269/ajtmh.2001.65.690

[B6] de Vries PJ Dien TK Clinical pharmacology and therapeutic potential of artemisinin and its derivatives in the treatment of malaria Drugs 1996 52 818 836 10.2165/00003495-199652060-00004 8957153

[B7] Ittarat W Pickard AL Rattanasinganchan P Wilairatana P Looareesuwan S Emery K Low J Udomsangpetch R Meshnick SR Recrudescence in artesunate-treated patients with falciparum malaria is dependent on parasite burden not on parasite factors Am J Trop Med Hyg 2003 68 147 152 12641403

[B8] Kyle DE Webster HK Postantibiotic effect of quinine and dihydroartemisin on *Plasmodium falciparum in vitro*: implications for a mechanism of recrudescence XIVth International Congress for Tropical Medicine and Malaria: abstract 0-22-6 1996 Nagasaki

[B9] Hoshen MB Na-Bangchang K Stein WD Ginsburg H Mathematical modelling of the chemotherapy of Plasmodium falciparum malaria with artesunate: postulation of 'dormancy', a partial cytostatic effect of the drug, and its implication for treatment regimens Parasitology 2000 121 237 246 10.1017/S0031182099006332 11085244

[B10] Teuscher F Gatton ML Chen N Peters J Kyle DE Cheng Q Artemisinin induced dormancy in *Plasmodium falciparum*: Duration, recovery rates and implications in treatment failure J Infect Dis 2010 202 1362 1368 10.1086/656476 20863228 PMC2949454

[B11] Witkowski B Lelievre J Lopez Barragan MJ Laurent V Su XZ Berry A Benoit-Vical F Increased tolerance to artemisinin in Plasmodium falciparum is mediated by a quiescence mechanism Antimicrob Agents Chemother 2010 54 1872 1877 10.1128/AAC.01636-09 20160056 PMC2863624

[B12] Bwijo B Hassan Alin M Wernsdorfer W Björkman A Efficacy of artemisinin and mefloquine combinations against *Plasmodium falciparum*. *In vitro *simulation of *in vivo *pharmacokinetics Trop Med Int Health 1997 2 461 467 10.1111/j.1365-3156.1997.tb00168.x 9217701

[B13] Nakazawa S Kanbara H Aikawa M Plasmodium falciparum: recrudescence of parasites in culture Exp Parasitol 1995 81 556 563 10.1006/expr.1995.1149 8542997

[B14] Nakazawa S Maoka T Uemura H Ito Y Kanbara H Malaria parasites giving rise to recrudescence in vitro Antimicrob Agents Chemother 2002 46 958 965 10.1128/AAC.46.4.958-965.2002 11897575 PMC127107

[B15] Thapar MM Gil JP Bjorkman A *In vitro *recrudescence of *Plasmodium falciparum *parasites suppressed to dormant state by atovaquone alone and in combination with proguanil Trans R Soc Trop Med Hyg 2005 99 62 70 10.1016/j.trstmh.2004.01.016 15550263

[B16] Gatton ML Cheng Q Investigating antigenic variation and other parasite-host interactions in Plasmodium falciparum infections in naive hosts Parasitology 2004 128 367 376 10.1017/S0031182003004608 15151141

[B17] Ahorlu CK Koram KA Ahorlu C de Savigny D Weiss MG Socio-cultural determinants of treatment delay for childhood malaria in southern Ghana Trop Med Int Health 2006 11 1022 1031 10.1111/j.1365-3156.2006.01660.x 16827703

[B18] Ndyomugyenyi R Magnussen P Clarke S Malaria treatment-seeking behaviour and drug prescription practices in an area of low transmission in Uganda: implications for prevention and control Trans R Soc Trop Med Hyg 2007 101 209 215 10.1016/j.trstmh.2006.06.004 16950487

[B19] Van Nam N de Vries PJ Van Toi L Nagelkerke N Malaria control in Vietnam: the Binh Thuan experience Trop Med Int Health 2005 10 357 365 10.1111/j.1365-3156.2005.01387.x 15807800

[B20] Borrmann S Adegnika AA Missinou MA Binder RK Issifou S Schindler A Matsiegui PB Kun JF Krishna S Lell B Kremsner PG Short-course artesunate treatment of uncomplicated Plasmodium falciparum malaria in Gabon Antimicrob Agents Chemother 2003 47 901 904 10.1128/AAC.47.3.901-904.2003 12604519 PMC149309

[B21] Menard D Matsika-Claquin MD Djalle D Yapou F Manirakiza A Dolmazon V Sarda J Talarmin A Association of failures of seven-day courses of artesunate in a non-immune population in Bangui, Central African Republic with decreased sensitivity of Plasmodium falciparum Am J Trop Med Hyg 2005 73 616 621 16172492

[B22] Saralamba S Pan-Ngum W Maude RJ Lee SJ Tarning J Lindegardh N Chotivanich K Nosten F Day NP Socheat D White NJ Dondorp AM White LJ Intrahost modeling of artemisinin resistance in Plasmodium falciparum Proc Natl Acad Sci USA 2011 108 397 402 10.1073/pnas.1006113108 21173254 PMC3017155

