# Supplementary material for: Artemisinin-induced parasite dormancy: a plausible mechanism for treatment failure
Source: Malar J. 2011 Mar 8;10:56. doi: 10.1186/1475-2875-10-56 (PMC3060839; doi:10.1186/1475-2875-10-56)
Supplement: Additional file 1 — Details of within-host model of P. falciparum infection in naïve host. [file 1475-2875-10-56-S1.PDF]

Additional material file 1: Details of within-host model of *P. falciparum* infection in naïve host

The within-host dynamics model mimics a *P. falciparum* infection within a malaria naïve host. The model was first reported by Gatton et al [1] where it was fit to data characteristic of the infection dynamics in people infected with the El Limon and Santee Cooper strains of *P. falciparum*.

Definitions

X_i : the number of ring stage parasites expressing PfEMP1 variant *i*.

$X = \sum_{i=1}^{60} X_i$: the total number of ring stage parasites.

Y : the number of mature (non-ring) parasites.

A_i : the amount of antibody against PfEMP1 variant *i*.

F: the magnitude of the non-specific immune response (referred to as fever).

T: fever threshold (number of parasites required to trigger non-specific immune response)

AT_i : binary variable indicating the presence/absence of antibody to PfEMP1 variant *i*.

AT: binary variable indicating the presence/absence of antibody to any PfEMP1 variant.

t: number of days since start of blood stage infection.

s_i: day that AT_i is set to true.

Establishing the initial infection

A blood-stage infection in the host commences with the release of 40,000 genetically identical merozoites from the liver (day 1),

$$X_i = \begin{cases} 40000 & i = 1 \\ 0 & 2 \leq i \leq 60 \end{cases}.$$

The parasites have a 2 day replication cycle: the parasites are assumed to be ring stage for the first 24 hours, followed by 24 hours as non-rings. The infection is assumed to cease when the number of parasites decreases below one.

Antibodies

The anti-PfEMP1 antibody response is produced after the number of parasites expressing a specific variant exceeds a threshold [2] and the corresponding time lag has elapsed. For the primary stimulation, antibodies are produced 7 days after $X_i >$ 6×10^7. For subsequent stimulation, antibodies are produced 2 days after X_i $>$ 3×10^7. AT_i is set to true once these conditions are met. When the antibody load has reduced to less than 1 for the triggered antibodies then the variant antibody test is reset to false (AT_i = false). **AT** is set to true (**AT** = TRUE) if AT_i is true for any *i* otherwise **AT** = FALSE.

An exponential function is used to describe the amount of anti-PfEMP1 antibody in the host (A_i), with the magnitude of the antibody response being dependent on whether the specific antibody has been previously produced. The model assumed limited cross-reactivity between anti-PfEMP1 antibodies [2].

If **AT**=FALSE then $A_i = 1.0, \quad \forall i.$

If **AT**=TRUE then $A_i = \begin{cases} \left(\frac{t-s_i}{30}\right) C_i e^{-\left(B_i \sqrt{(t-s_i)/30}\right)}, & AT_i \text{ is true,} \\ 1.05, & AT_i \text{ is false.} \end{cases}$

where $C_i = \begin{cases} N(10000\,, 500), & \text{primary stimulation,} \\ N(35000\,, 500), & \text{otherwise.} \end{cases}$ and $B_i = \begin{cases} N(3.5\,, 0.1), & \text{primary stimulation,} \\ N(3.0\,, 0.1)\,, & \text{otherwise.} \end{cases}$

PfEMP1 switching

To mimic the process of antigenic variation in *Pf*EMP1, the model assumes that each parasite has a repertoire of *var* genes encoding 60 PfEMP1 variants and used the switching process hypothesized by Gatton *et al.* [3].

To implement the switching process it is assumed that (a) all parasites express the same *var* gene when released from the liver, (b) once parasites switch away from this initial *var* gene they are unable to switch back to it, and (c) the switch rates for all individual *var* genes are pre-determined. Switching is implemented immediately prior to replication so that all daughter merozoites express the same PfEMP1 variant as the parent parasite. The switching probability to variant *i* is

$$\begin{aligned} &P_1 = 0.0, \\ &P_i = B^{(i-2)}, && 2 \le i \le 12, \\ &P_{13} = 10^{-7} P_{12}, && \\ &P_i = P_{13} C^{(i-13)}, && 14 \le i \le 60, \end{aligned} \quad ,$$

where $B = 1.3^{-8/10}$ and $C = 1.18^{-39/47}$ [4].

The number of parasites switching from PfEMP1 variant *i* to variant *j* is $S_{i,j} \sim Bi\left(X_i, \frac{0.18 \times P_j}{\sum_{k=1}^{60} P_k}\right)$.

Non-specific immune response

The numerical value representing the non-specific immune response (*F*) is constrained to be between 0 and 5 and is related to the number of parasites replicating on the specified day:

$$F = \begin{cases} 0, & X \le T, \\ \dfrac{X-T}{20T}, & T < X \le 101T, \\ 5, & X > 101T. \end{cases}$$

where the fever threshold, *T*, is computed as

$$T = \begin{cases} 5\times 10^{6} \times 10^{0.271t+1.6846+N(0,0.3)}/3, & t \le 6, \\ 5\times 10^{6} \times 10^{3.339+N(0,0.3)}/3, & t > 6. \end{cases}.$$

The non-specific immune response has the effect of reducing the number of ring-stage parasites to $X_i = Bi\left(X_i, e^{-F}\right)$, and the number of mature parasites to $Y = Bi\left(Y, e^{-2F}\right)$ [5].

Treatment and dormancy

Treatment was triggered once *F* > 0.35. When treatment was administered it was assumed that a proportion of ring-stage parasites and mature stage parasites became dormant, with the remainder being killed. Those parasites which became dormant did not replicate during dormancy, nor were they subject to anti-PfEMP1 antibodies or any other host immune response. The parasites wake from dormancy following the specified time-dependent profile to resume normal growth and immune susceptibility.

Replication

During the replication step some parasites convert to gametocytes while the remainder replicate. The number of gametocytes expressing PfEMP1 variant *i* produced at each replication cycle (G_i)

was calculated by applying a conversion rate, based on a log-normal distribution, to the number of parasites, immediately after replication, as previously described [6]. For the purposes of this investigation, once the ring stage parasites converted to gametocytes they were no longer of interest.

The host clonal immune response acts to reduce the number of merozoites that survive one complete replication cycle, causing a reduction in the effective growth rate of the parasites:

$$X_i = Bi\left(X_i \times growth, \frac{1-K_i}{A_i} \right)$$

where $K_i = 0.2 + \frac{0.45}{1+e^{(30-t)/6}}$,and *growth* is 16 [4].

References

1. Gatton ML, Cheng Q: **Investigating antigenic variation and other parasite-host interactions in Plasmodium falciparum infections in naive hosts**. *Parasitology* 2004, **128**(Pt 4):367-376.
2. Krause DR, Gatton ML, Frankland S, Eisen DP, Good MF, Tilley L, Cheng Q: **Characterization of the antibody response against Plasmodium falciparum erythrocyte membrane protein 1 in human volunteers**. *Infect Immun* 2007, **75**(12):5967-5973.
3. Gatton ML, Peters JM, Fowler EV, Cheng Q: **Switching rates of Plasmodium falciparum var genes: faster than we thought?** *Trends Parasitol* 2003, **19**(5):202-208.
4. Gatton ML, Cheng Q: **Modeling the development of acquired clinical immunity to Plasmodium falciparum malaria**. *Infect Immun* 2004, **72**(11):6538-6545.
5. Kwiatkowski D: **Febrile temperatures can synchronize the growth of *Plasmodium falciparum* in vitro**. *J Exp Med* 1989, **169**:357-361.

6. Gatton ML, Cheng Q: **Plasmodium falciparum infection dynamics and transmission potential following treatment with sulfadoxine-pyrimethamine**. *J Antimicrob Chemother* 2006, **58**(1):47-51.
